# Crossed McMurry Coupling Reactions for Porphycenic Macrocycles: Non-Statistical Selectivity and Rationalisation

**DOI:** 10.1002/ejoc.201500221

**Published:** 2015-04-29

**Authors:** Thomas Y Cowie, Lorna Kennedy, Justyna M Żurek, Martin J Paterson, Magnus W P Bebbington

**Affiliations:** [a] School of EPS – Institute of Chemical Sciences, Heriot-Watt University Riccarton, Edinburgh, EH14 4AS, UK E-mail: m.j.paterson@hw.ac.uk m.w.p.bebbington@hw.ac.uk http://www.theophotochem.eps.hw.ac.uk/Home.html, http://www.hw.ac.uk/schools/engineering-physical-sciences/staff-directory/mb.htm

**Keywords:** Porphycenes, Macrocycles, McMurry coupling, Heterocyclic chemistry, Photochemistry, Redox chemistry

## Abstract

Crossed McMurry reactions of bifuran- or bithiophenedicarbaldehydes with bipyrroledicarbaldehydes have been studied for the first time. Only those porphycenic macrocycles derived from homocoupled McMurry products were formed. The results are explained by using both density functional theory and electron propagator computations to model the electron affinity of the dialdehyde starting materials. It was predicted that bifuran\bithiophene cross-coupling would indeed occur, and this was demonstrated by the first synthesis of a novel dioxa,dithio hetero-porphycenoid annulene. This approach will allow the prior identification of viable substrates for related crossed McMurry reactions.

## Introduction

**Figure 1:**
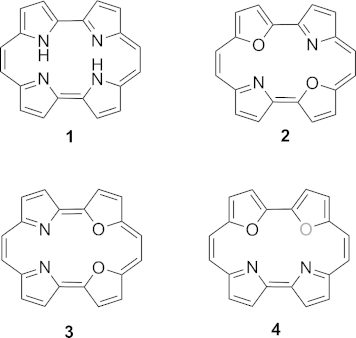
Structure of the parent porphycene **1** and dioxaporphycene isomers **2**–**4**.

Porphycenes are structural isomers of porphyrins, consisting of a planar macrocyclic ring with an aromatic 18π-electron configuration (Figure 1). Their chemistry has been widely studied,[1] and they have attracted considerable interest for their potential applications in catalysis,[2] materials chemistry,[3] non-linear optics,[4] photo-inactivation of bacteria[5] and protein mimicry.[6] In particular, they have recently become of interest as sensitisers for two-photon absorption (TPA) for use in photodynamic therapy (PDT).[7] In PDT one needs to photosensitise in the tissue transparency window (600–1000 nm), and porphyrin chromophores have been the standard for linear absorption. However, their maximum absorption at 630 nm limits the use due to e.g., tissue depth penetration. The idea of using two photons, each of twice the excitation wavelength is a promising approach in modern PDT research. Unfortunately, the TPA characteristics of porphyrins are not ideal (i.e., low TPA cross section at desired wavelengths). However, recent work has shown that their structural isomers, the porphycenes do have this desired property.[8a,8b] As part of an interdisciplinary research program aimed at the rational design of two-photon sensitisers, we have previously undertaken a computational study on the effect of heteroatom substitution on TPA properties.[9a,9b] It has been predicted that – while one-photon absorption is insensitive to core aromatic substitution, and results in almost identical absorption spectra (i.e., Soret and Q bands) for such electronic isomers – crucially the non-linear (two-photon) absorption is highly sensitive to such features. This fascinating aspect highlights the very subtle molecular tuning that is possible for non-linear optical applications. Here, incorporation of two oxygen atoms into the porphycene core (Figure 1) was predicted to lead to resonance enhancement (in the Q-band region) that produced a remarkable improvement in the TPA cross-section. Only a single macrocycle containing two pyrrole and two furan units (of type **2**) has been reported, and no yield or characterisation data are disclosed.[10]

**Scheme 1:**
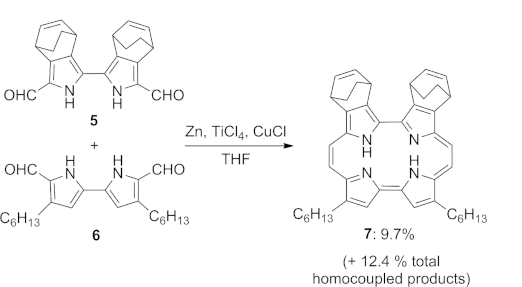
Recent porphycene synthesis by crossed McMurry reaction.

**Scheme 2:**
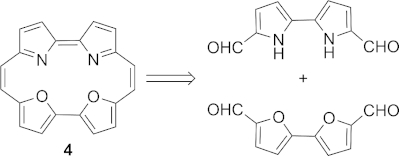
Retrosynthesis of dioxaporphycene **4**.

Porphycenes are traditionally prepared by reductive McMurry coupling of two bipyrroldicarbaldehyde units in modest yield.[1,11] Very few other reliable methods exist for their preparation.[12a,12b] Examples of peripheral functionalisation and the inclusion of additional heteroatoms within the ring structure have emerged.[13]–[16] Cross-coupling of two different bipyrroles has also been reported (Scheme 1).[17] We reasoned that one of the most promising dioxaporphycene systems for TPA, namely **4**, might be accessed most directly through crossed McMurry coupling of bifuran and bipyrrole subunits (Scheme 2). Typically, statistical mixtures are obtained in crossed McMurry reactions.[18] We therefore chose to carry out a systematic study of these processes using mixtures of furan, pyrrole and thiophene-derived dicarbonyl substrates relevant to our desired targets, which have until now been unexplored.

## Results and Discussion

Bipyrrole[17i] **6** and bifuran[19] **9** (Table 1) were prepared according to literature methods, and an equimolar mixture of these dialdehydes was subjected to standard McMurry conditions for porphycene synthesis. Intriguingly, this reaction returned only macrocycles derived from homocoupled products **11b** and **12a** in a 1:3 ratio, with no signals in the crude NMR spectrum indicating the formation of any other macrocyclic products such as **13**.[20] The total yield of macrocyclic products was within the range of what would normally be expected for a porphycene synthesis,[1] suggesting that we had successfully isolated all the macrocycles present. Our initial, tentative explanation for this, based on the accepted mechanism for the McMurry reaction,[18] was that the more electron-rich pyrrole was both more difficult to reduce *and* less reactive in the subsequent dimerisation of the radical anion species than the bifuran. Under these circumstances the bifuran would be consumed much more rapidly than the bipyrrole, leading to the observed selectivity. Use of diacetylbifuran[21] **10** was attempted in order to reduce the rate of homodimerisation, but once again only the homodimeric products **11b** and **12b** were obtained, albeit in a 2:3 ratio. To confirm that the pyrrole components we had used were compatible with the reaction conditions, we successfully prepared the mixed porphycene **11c** from **6** and **8**, obtained as part of what appears to be a statistical mixture including homocoupled products **11a** and **11b**.

**Table 1:** Crossed McMurry reactions between biheterocycle subunits.[a]

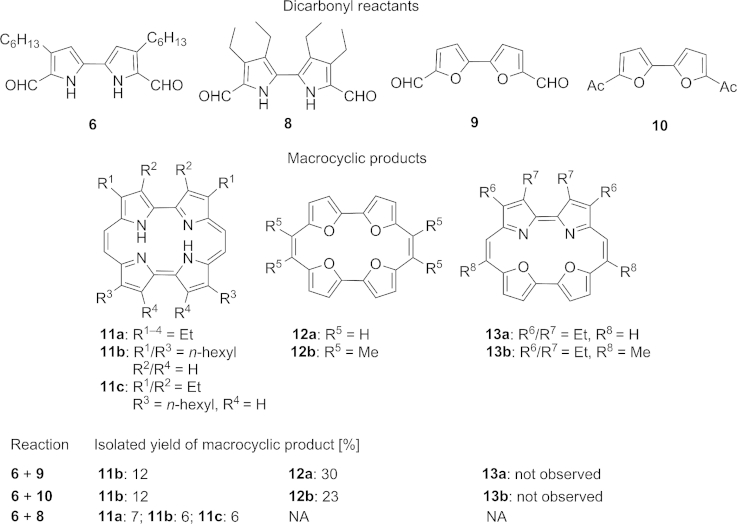

[a] Macrocycles **11a**, **11b**, **12a** and **12b** were prepared independently by homocoupling of the appropriate dialdehydes; see Experimental Section and Supporting Information. Diagnostic signals for macrocyclic products were found between *δ* = 6 and 9 ppm in the ^1^H NMR spectra.

The success of crossed McMurry reactions is known to depend in part on the reducibility of the reactants.[18,22] We postulated that for crossed McMurry reactions to be successful in macrocycle synthesis, each compatible coupling partner should have a similar rate of reduction, so that each reduced species can exist simultaneously at a significant concentration. It was supposed that single electron transfer (SET) from the reducing metal was likely to be the rate-determining step in the McMurry reaction. We therefore computed the electron affinity, and thus overall reducibility, of each biheterocycle system, in two complementary ways. Firstly, we used electron propagator theory (P3 propagator method) to calculate the 3rd-order correlated electron affinity (EA) for the LUMO (using the cc-pVTZ basis).[23a]–[23c] These EAs are given in Table 2. Notably the pole strengths (PS) are all above 0.85, and thus the quasi-single particle picture is valid. Density functional theory (B3LYP functional and cc-pVTZ basis) was used to directly calculate relaxed electron affinities (difference between geometry-optimised neutral and radical anions, i.e., opposite sign electron attachment energies). We omit the hexyl chains but note that calibration calculations with smaller basis sets show the effect of such pyrrole-substituted alkyl chains to be small.

**Table 2:** McMurry reaction coupling partners, propagator-derived LUMO electron affinity (EA) and B3LYP relaxed electron affinity (PS = pole strength)

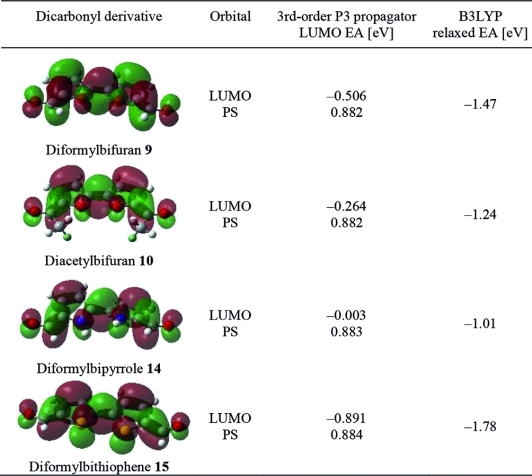

Thus, the 3rd-order LUMO EA is a key parameter related to the kinetics of electron transfer from the metal, and thus the rate of formation of the key radical anion species, as it relates to the energetic requirements for the initial electron transfer. The relaxed EA from DFT relates to the stability of such radical anions once formed. We note that the same trend is seen for both approaches.

The parent bipyrrole system **14**, (used here without pendant groups for conformational simplicity) with a LUMO EA of –0.003 eV, is therefore significantly more difficult to reduce than the bifurans and bithiophenes under study, which have much more favorable LUMO EAs ranging from –0.26 to –0.89 eV. These results are consistent with SET to the bifurans **9** and **10** being therefore more rapid than for the bipyrroles, which could explain the complete selectivity for the formation of homodimerised products in their attempted crossed McMurry reactions.

**Scheme 3:**
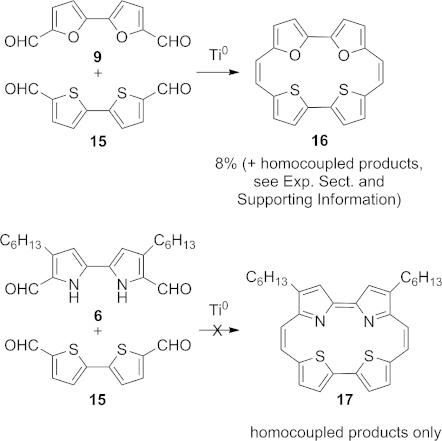
Crossed McMurry reaction of bithiophene **15**.

We observed that the LUMO EAs for the bifurandicarbaldehyde **9** and the bithiophenedicarbaldehyde[24] **15** were both significantly more negative than those for the bipyrroles **6** and **8**. Thus, they could be potential reaction partners in a crossed McMurry reaction, and we indeed found that a mixture of **9** and **15** gave some of the heterodimer-derived macrocycle **16** in addition to homodimerised products[25] (Scheme 3).

**Figure 2:**
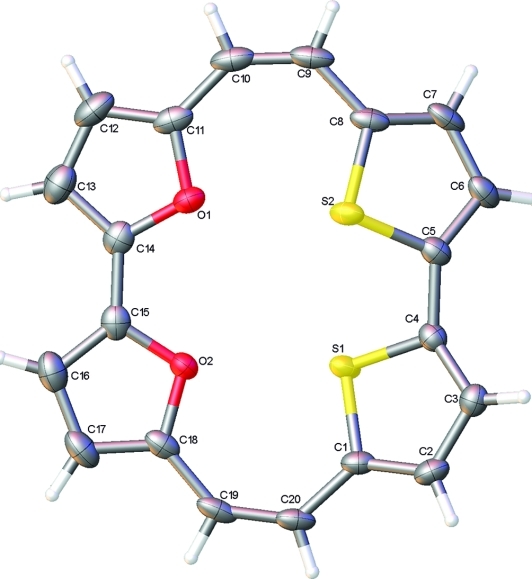
X-ray structure of **16** (ellipsoids at the 50 % level).

Careful chromatography allowed the isolation of the new macrocyclic system **16**, and crystals were obtained for an X-ray diffraction study to confirm the structure[26] (Figure 2). As expected, the macrocycle is not planar, as it cannot readily aromatise to an 18π-electron system. The furan rings appear to be in conjugation with the C=C double bonds (the O1–C11–C10–C9 dihedral angle is only 5.0°), but the more bulky thiophene rings are rotated out of the ring plane more significantly (S2–C8–C9–C10 dihedral angle 24.9°). The solution-phase NMR spectroscopic data also do not indicate the presence of any ring currents (see Experimental Section).

As a further control experiment, we also subjected a mixture of bipyrrole and bithiophene components **6** and **15** to the standard reaction conditions, and once again observed only products from homocoupling. It would appear that the reducibility of the dialdehyde components is not the only parameter affecting the relative rates of product formation from homo- and heterodimerisation, since the proportions of the products do not link in a simple way to the relative values for the LUMO EA. Steric effects are also likely to play a role in the C–C bond-forming step, and thiophene is known to be significantly more bulky than furan or pyrrole.[27] However, given that the bithiophene is known to homocouple,[13b,^28^] and that formation of porphycenes **11a**–**c** involves more steric demand than dioxaporphycenes **13a**–**b**, it seems unlikely that a purely steric explanation accounts for the selectivity we have observed. Additionally, preliminary semiempirical PM6 heats of formation calculations predict that **4** is more stable than **1**. It is therefore likely that the EA of the starting materials remains an important factor in governing the selectivity of these crossed McMurry reactions.

## Conclusions

We have examined the unusual product distributions in crossed McMurry reactions of biheterocycledicarbaldehydes and correlated the results with calculated electron affinities, as a measure of reducibility, of the starting materials. Work is currently underway to prepare bipyrrole and bifuran/bithiophene partners with more closely matched electron affinities, to test further the hypothesis presented herein. This approach will result in the rational design of suitable components for crossed McMurry reactions, ultimately allowing access to unsymmetrical porphycenes for further study.

## Experimental Section

**General:** Chemical shifts (*δ* in ppm) were referenced to tetramethylsilane (TMS) or to residual solvent peaks (CDCl_3_: *δ*H = 7.26 ppm); *J* values are given in Hz, and s, br. s, d, dd, ddd, dt, t, br. t, td, q, quint, sext and m abbreviations correspond to singlet, broad singlet, doublet, doublet of doublet, doublet of doublet of doublets, doublet of triplets, triplet, broad triplet, triplet of doublets, quartet, quartet of triplets, quintet, sextet and multiplet, respectively. IR spectra were obtained deposited neat or as a chloroform solution to a diamond/ZnSe plate. CCDC-1038696 (for **12b**), -CCDC-1038697 (for **11c**), and -CCDC-1038698 (for **16**) contain the supplementary crystallographic data for this paper. These data can be obtained free of charge from The Cambridge Crystallographic Data Centre via www.ccdc.cam.ac.uk/data_request/cif.

**Typical Procedure for the Preparation of Porphycenic Macrocycles from Diformylbiheterocycles:** Under nitrogen, a suspension of activated zinc (3.7 g) and copper(I) chloride (222 mg, 2.22 mmol) in THF (100 mL), was added titanium tetrachloride (3.11 mL, 28.8 mmol) dropwise. Upon completion of addition, the solution was stirred at reflux for 2 h. To the refluxing mixture a solution of **6** (185 mg, 0.52 mmol) and **8** (156 mg, 0.52 mmol) in THF (100 mL) was added dropwise over 1 h. The solution was stirred for an additional 1 h. The solution was cooled to 0 °C and quenched by the dropwise addition of ammonium hydroxide solution (6 %, 100 mL) over 30 min. The solution was extracted with DCM (200 mL) and the organic layer separated and dried (Na_2_SO_4_). After removal of the solvent under reduced pressure, the residue was chromatographed, and the target compounds were isolated to yield **11a** (10 mg, 7 %), **11b** (11 mg, 6 %), **11c** (17 mg, 6 %).

**2,3,6,7,12,13,16,17-Octaethylporphycene (11a):**[28] Blue crystals (10 mg, 7 %); m.p. 212–214 °C. ^1^H NMR (400 MHz, CDCl_3_): *δ* = 9.49 (s, 4 H), 4.03 (q, *J* = 7.5 Hz, 8 H), 3.88 (q, *J* = 7.6 Hz, 8 H), 1.66 (t, *J* = 7.6 Hz, 12 H), 1.59 (t, *J* = 7.5 Hz, 12 H) ppm. ^13^C NMR (101 MHz, CDCl_3_): *δ* = 143.4, 142.2, 137.4, 136.9, 110.0, 29.9, 21.6, 20.1, 18.2 ppm. IR 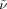 = 2961, 2920, 2867, 1534, 1518, 1449, 1371, 1301, 1192, 1029 cm^–1^.

**2,7,12,17-Tetrahexylporphycene (11b):**[17] Violet crystals (11 mg, 6 %); m.p. 120–122 °C. ^1^H NMR (400 MHz, CDCl_3_): *δ* = 9.79 (s, 4 H), 9.36 (s, 4 H), 4.10 (t, *J* = 7.6 Hz, 8 H), 3.29 (s, 2 H), 2.44 (m, 8 H), 1.82 (m, 8 H), 1.59 (m, 8 H), 1.48 (m, 8 H), 1.00 (t, *J* = 7.3 Hz, 12 H) ppm. ^13^C NMR (101 MHz, CDCl_3_): *δ* = 145.1, 143.6, 134.2, 122.6, 110.5, 32.1, 29.7, 28.5, 22.8, 14.2 ppm. IR 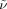 = 2950, 2923, 2852, 1697, 1558, 1454, 1367, 1298, 1259, 1233, 1206, 1090, 1033, 1009 cm^–1^.

**12,13,16,17-Tetraethyl-2,7-dihexylporphycene (11c):** Deep blue crystals (17 mg, 6 %); m.p. 133–135 °C. ^1^H NMR (400 MHz, CDCl_3_): *δ* = 9.69 (d, *J* = 11.2 Hz, 2 H), 9.62 (d, *J* = 11.2 Hz, 2 H), 9.26 (s, 2 H), 4.10 (q, *J* = 7.5 Hz, 4 H), 4.03 (t, *J* = 7.5 Hz, 4 H), 3.95 (q, *J* = 7.6 Hz, 4 H), 2.37 (m, 4 H), 1.75 (m, 4 H), 1.73 (t, *J* = 7.6 Hz, 6 H), 1.64 (t, *J* = 7.5 Hz, 6 H), 1.43 (m, 4 H), 1.26 (br. s, 2 H), 0.96 (t, *J* = 7.3 Hz, 6 H), 0.86 (m, 4 H) ppm. ^13^C NMR (101 MHz, CDCl_3_): *δ* = 145.0, 144.1, 143.3, 141.7, 137.3, 136.0, 135.0, 122.5, 110.6, 110.1, 32.0, 29.7, 28.4, 22.8, 22.6, 21.5, 20.1, 18.2, 18.1, 14.2 ppm. IR: 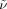 = 2961, 2922, 2853, 1504, 1467, 1449, 1370, 1292 cm^–1^. MS (FTMS + p NSI Full MS): calcd. for C_40_H_54_N_4_ [M + H]^+^ 590.4421; found 591.4413.

**(3*Z*,7*Z*)-1,2,5,6(2,5)-Tetrafuranacyclooctaphane-3,7-diene (12a):** Compound **12a** was obtained from the crossed McMurry reaction between **6** (100 mg, 0.28 mmol) and **9** (53 mg, 0.28 mmol). The crude oil was chromatographed on silica gel (CH_2_Cl_2_) to yield **11b** (11 mg, 12 %) and **12a** as dark red crystals (14 mg, 31 %); m.p. 270–271 °C. ^1^H NMR (400 MHz, C_6_D_6_): *δ* = 4.95 (d, *J* = 3.2 Hz, 4 H), 4.85 (d, *J* = 3.2 Hz, 4 H), 4.19 (s, 4 H) ppm. ^13^C NMR (100 MHz, C_6_D_6_): *δ* = 155.5, 149.3, 116.3, 133.9, 109.2 ppm. IR: 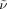 = *v* 3126, 1977, 1682, 1614, 1531, 1436, 1389, 1346, 1276, 1201, 1024 cm^–1^.

**(3*Z*,7*Z*)-3,4,7,8-Tetramethyl-1,2,5,6(2,5)-tetrafuranacyclooctaphane-3,7-diene (12b):** Compound **12b** was obtained from the crossed McMurry reaction between **6** (100 mg, 0.28 mmol) and **10** (61 mg, 0.28 mmol). The crude oil was chromatographed on silica gel [petroleum ether (40–60 °C)/EtOAc ca. 20 %] to yield **11b** (11 mg, 12 %) and **12b** as brown/orange crystals (12 mg, 23 %); m.p. 205–206 °C (dec.). ^1^H NMR (300 MHz, CDCl_3_): *δ* = 6.30 (d, *J* = 3.4 Hz, 4 H), 6.20 (d, *J* = 3.4 Hz, 4 H), 2.04 (s, 12 H) ppm. ^13^C NMR (75 MHz, CDCl_3_): *δ* = 155.8, 144.1, 122.9, 111.0, 109.5, 45.0, 29.6, 19.9 ppm. IR: 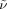 = 2942, 2864, 1444, 1376, 1280, 1113 cm^–1^. MS (FTMS + p NSI Full MS): calcd. for C_24_H_20_O_4_ [M + H]^+^ 373.1434; found 373.1433.

**(3*Z*,7*Z*)-1,2(2,5)-Difurana-5,6(2,5)-dithiophenacyclooctaphane-3,7-diene (16):** Compound **16** was obtained from the crossed McMurry reaction between **9** (128 mg, 0.68 mmol) and **15** (150 mg, 0.68 mmol). The crude oil was chromatographed on silica gel [petroleum ether (40–60 °C)/EtOAc ca. 5 %] to yield the expected[25] dimeric macrocycle **DI** (19 mg, 14 %; see the Supporting Information), trimeric macrocycle **TRI** (22 mg, 12 %; see the Supporting Information) as well as **12a** (17 mg, 16 %) and **16** as brown crystals (9 mg, 8 %). Data for **16**: m.p. 146–148 °C. ^1^H NMR (400 MHz, CDCl_3_): *δ* = 6.70 (d, *J* = 3.6 Hz, 2 H), 6.61 (ddd, *J* = 3.6, 0.9, 0.3 Hz, 2 H), 6.10 (d, *J* = 3.5 Hz, 2 H), 5.98 (dt, *J* = 3.4, 0.5 Hz, 2 H), 5.94 (ddd, *J* = 12.5, 0.9, 0.5 Hz, 2 H), 5.63 (d, *J* = 12.5 Hz, 2 H) ppm. ^13^C NMR (100 MHz, CDCl_3_): *δ* = 153.2, 146.5, 142.5, 139.0, 128.4, 124.8, 118.7, 118.5, 117.0, 111.6 ppm. IR: 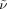 = 2927, 1651, 1603, 1537, 1452, 1391, 1260, 1017, 906, 795, 744 cm^–1^. MS (FTMS + p NSI Full MS): calcd. for C_20_H_12_O_2_S_2_ [M + H]^+^ 349.0351; found 349.0349.

**Attempted Formation of (9*Z*,19*Z*)-12,17-Dihexyl-21,22-dithiaporphyrin-9,19-diene (17):** Compound **17** was not obtained from the crossed McMurry reaction between **6** (100 mg, 0.28 mmol) and **15** (62 mg, 0.28 mmol). The crude oil was chromatographed on silica gel [petroleum ether (40–60 °C)/EtOAc ca. 20 %] to yield **11b** (7.1 mg, 8 %), the expected[25] dimeric macrocycle **DI** (2.1 mg, 4 %; see the Supporting Information) and trimeric macrocycle **TRI** (5.8 mg, 11 %; see the Supporting Information).

**Supporting Information** (see footnote on the first page of this article): ^1^H and ^13^C NMR spectra for all macrocycles, including annotated crude spectra of crossed reactions; X-ray crystallographic data for compounds **11c**, **12b** and **16**.

## Supporting Information

As a service to our authors and readers, this journal provides supporting information supplied by the authors. Such materials are peer reviewed and may be re-organized for online delivery, but are not copy-edited or typeset. Technical support issues arising from supporting information (other than missing files) should be addressed to the authors.

miscellaneous_information
