# Supplementary material for: Crossed McMurry Coupling Reactions for Porphycenic Macrocycles: Non-Statistical Selectivity and Rationalisation
Source: European J Org Chem. 2015 Apr 29;2015(17):3818–23. doi: 10.1002/ejoc.201500221 (PMC4502767; doi:10.1002/ejoc.201500221)
Supplement: Supplementary file 1 — miscellaneous_information [file ejoc2015-3818-sd1.pdf]

Eur. J. Org. Chem. **2015** · ISSN 1099–0682

SUPPORTING INFORMATION

DOI: 10.1002/ejoc.201500221
Title: Crossed McMurry Coupling Reactions for Porphycenic Macrocycles: Non-Statistical Selectivity and Rationalisation
Author(s): Thomas Y. Cowie, Lorna Kennedy, Justyna M. Żurek, Martin J. Paterson,* Magnus W. P. Bebbington*

^{1}H and ^{13}C NMR spectra for macrocyclic products and ^{1}H NMR spectra of crude reaction mixtures.

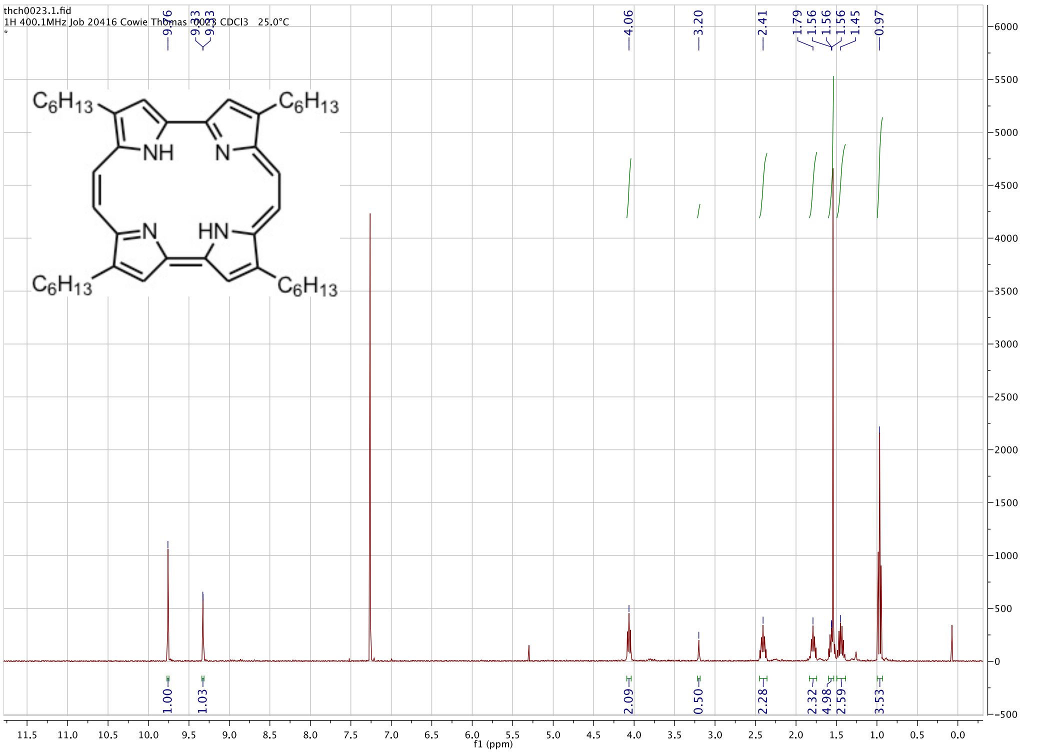

Figure 1. ^{1}H NMR Spectrum of 11b in $CDCl_3$

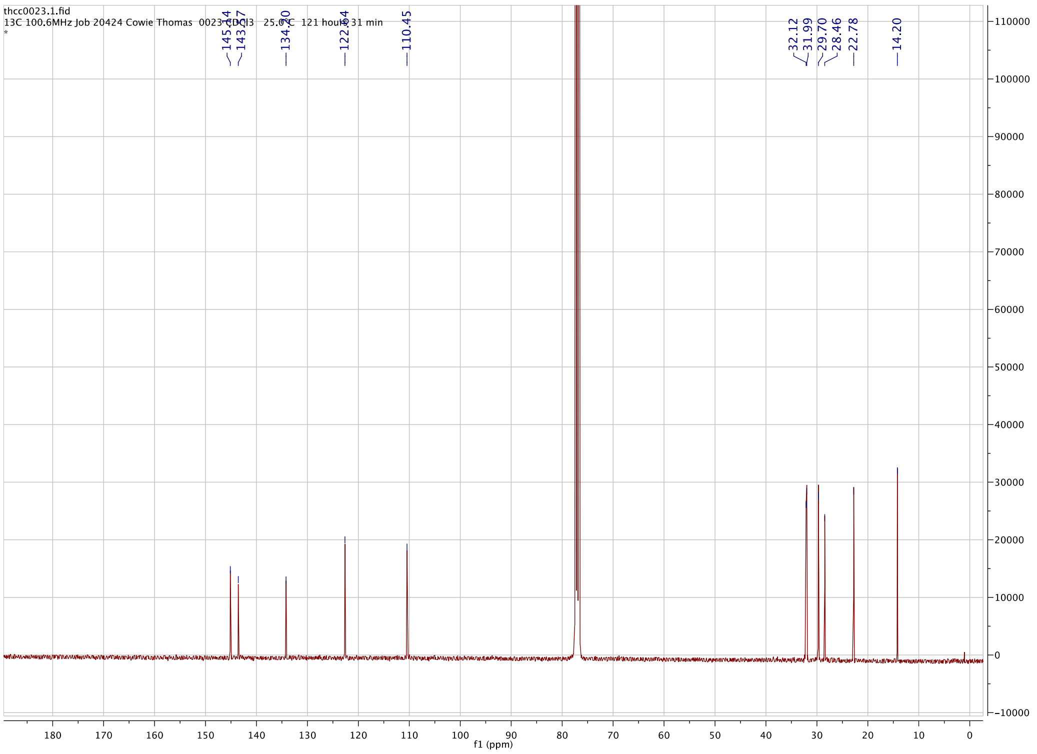

Figure 2. ^{13}C NMR Spectrum of 11b in $CDCl_3$

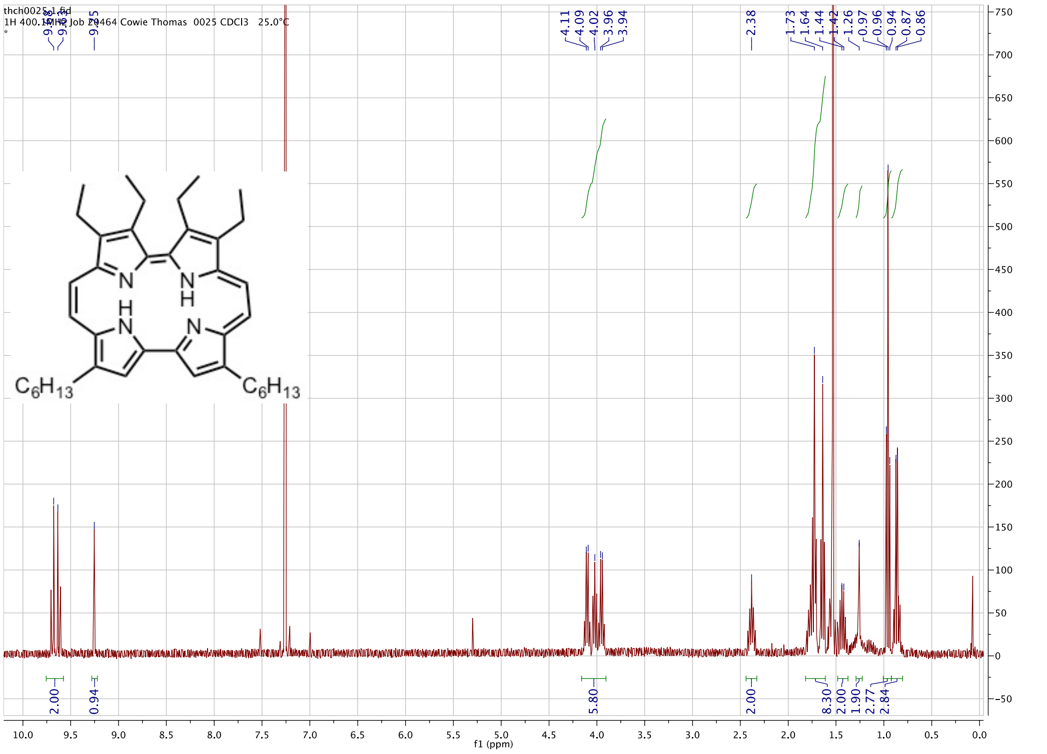

Figure 3. ^{1}H NMR Spectrum of 11c in $CDCl_3$

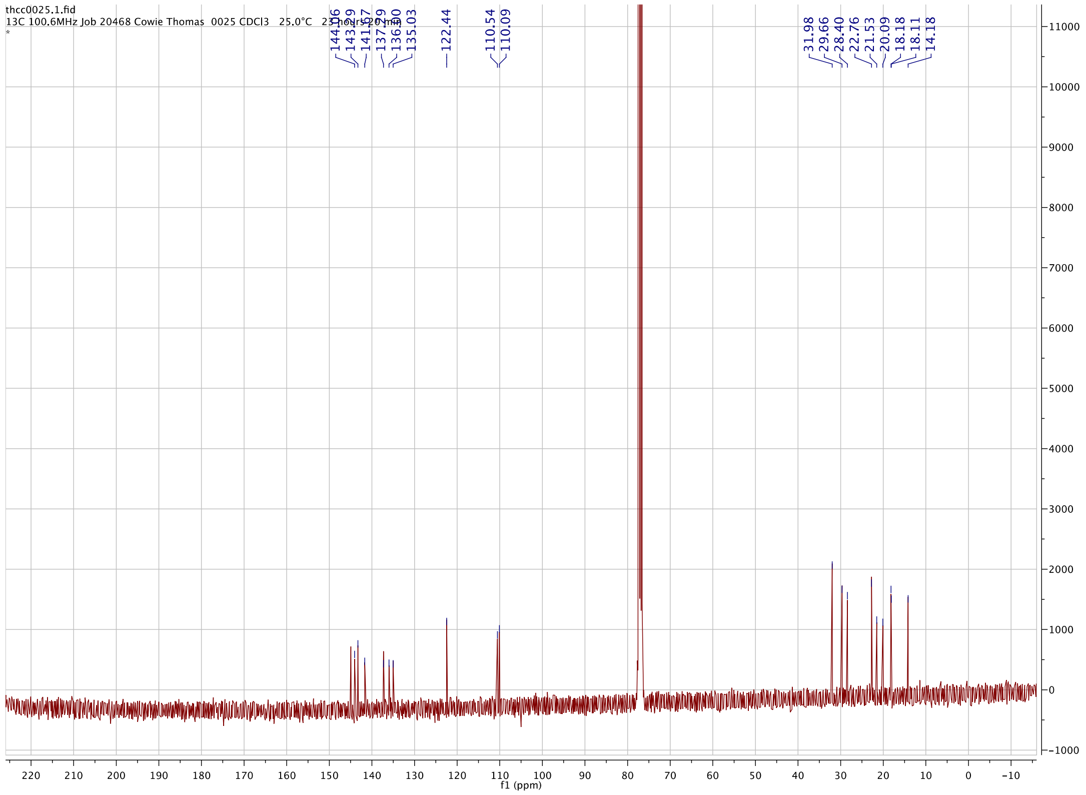

Figure 4. ^{13}C NMR Spectrum of 11c in $CDCl_3$

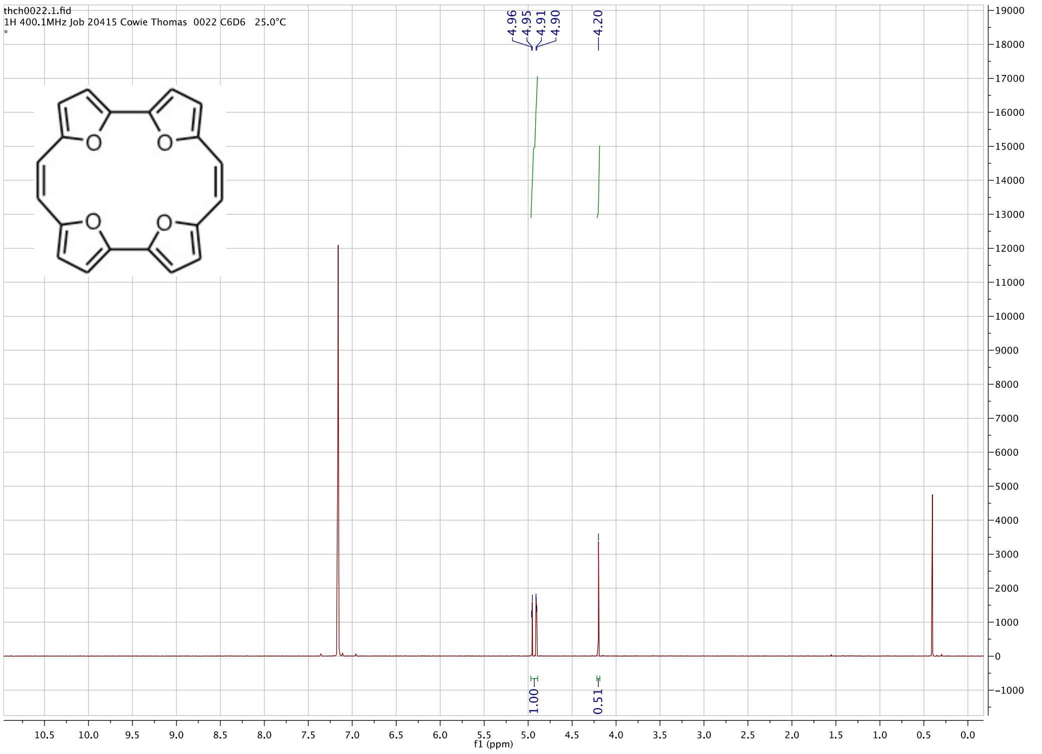

Figure 5. ^{1}H NMR Spectrum of 12a in C_6D_6

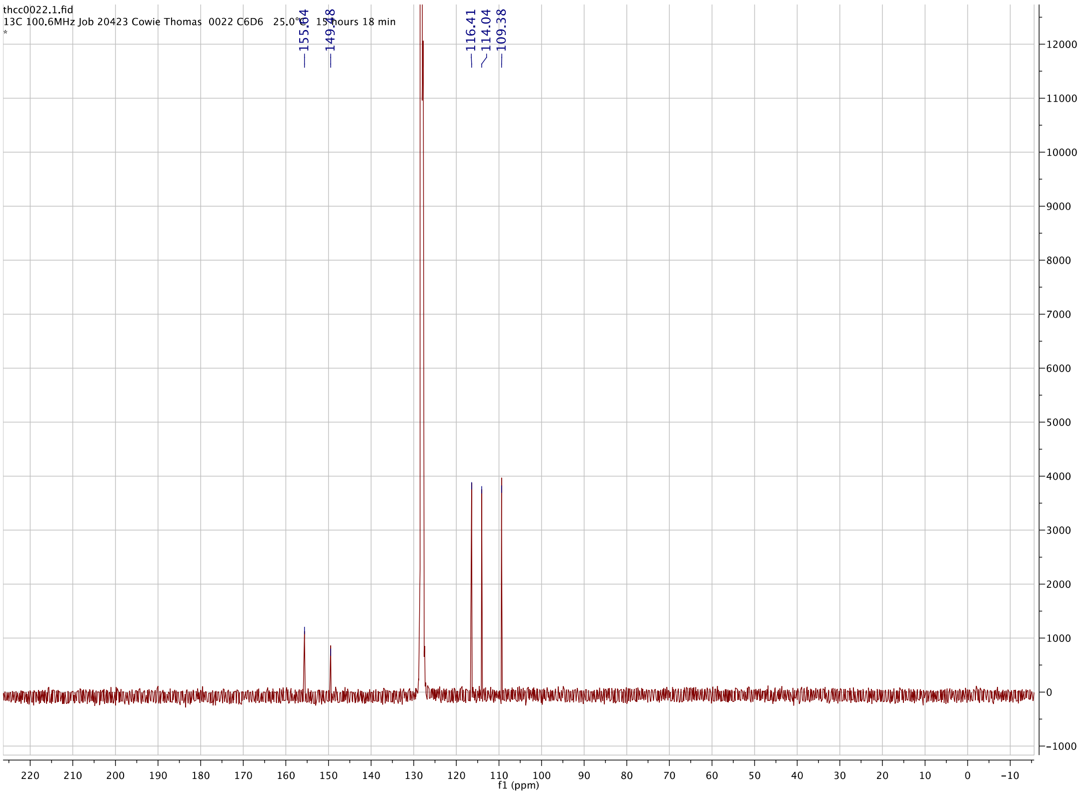

Figure 6. ^{13}C NMR Spectrum of 12a in C_6D_6

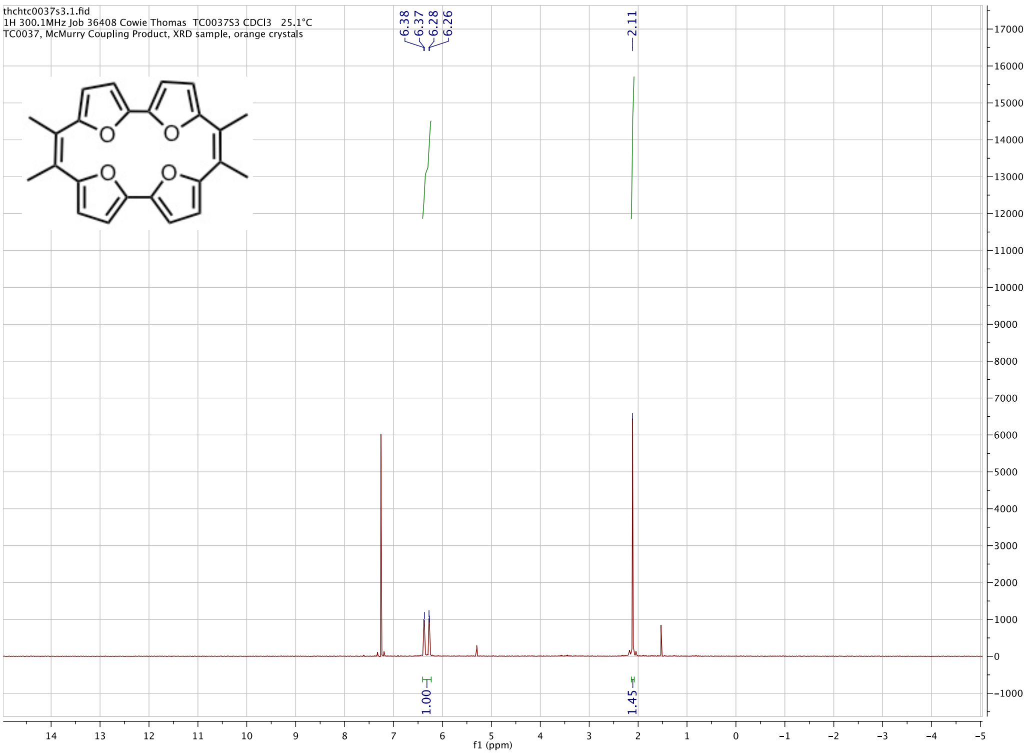

Figure 7. ^{1}H NMR Spectrum of 12b in $CDCl_3$

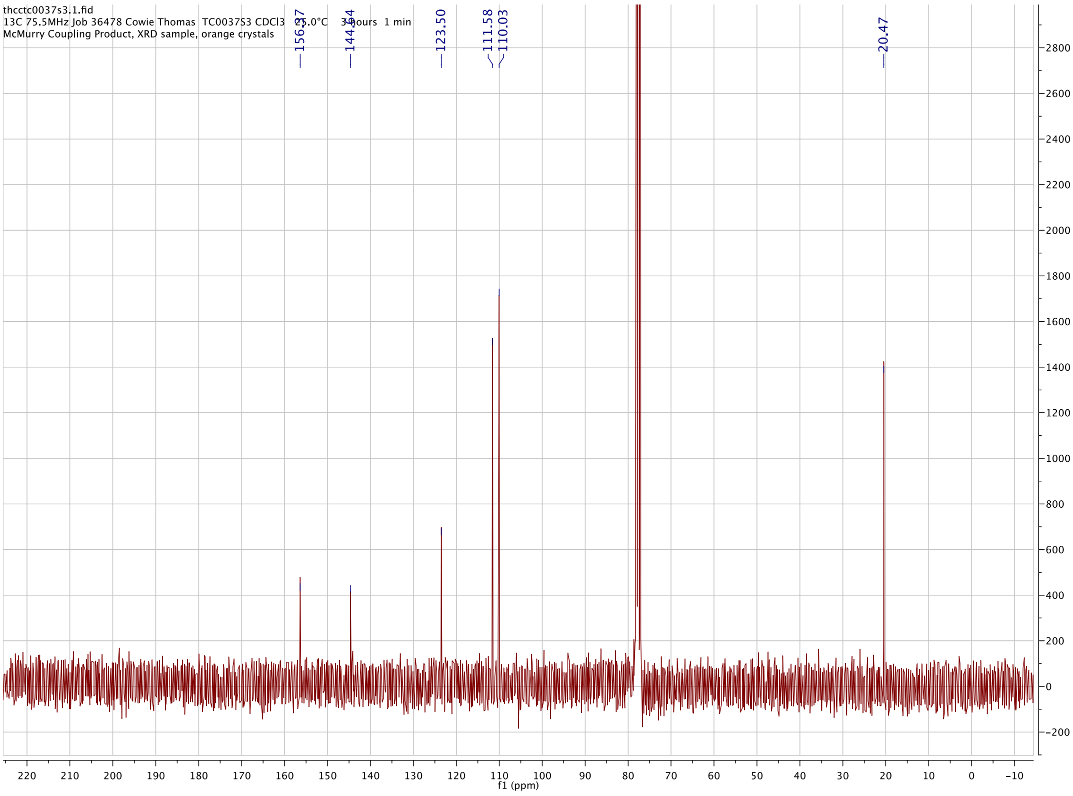

Figure 8. ^{13}C NMR Spectrum of 12b in $CDCl_3$

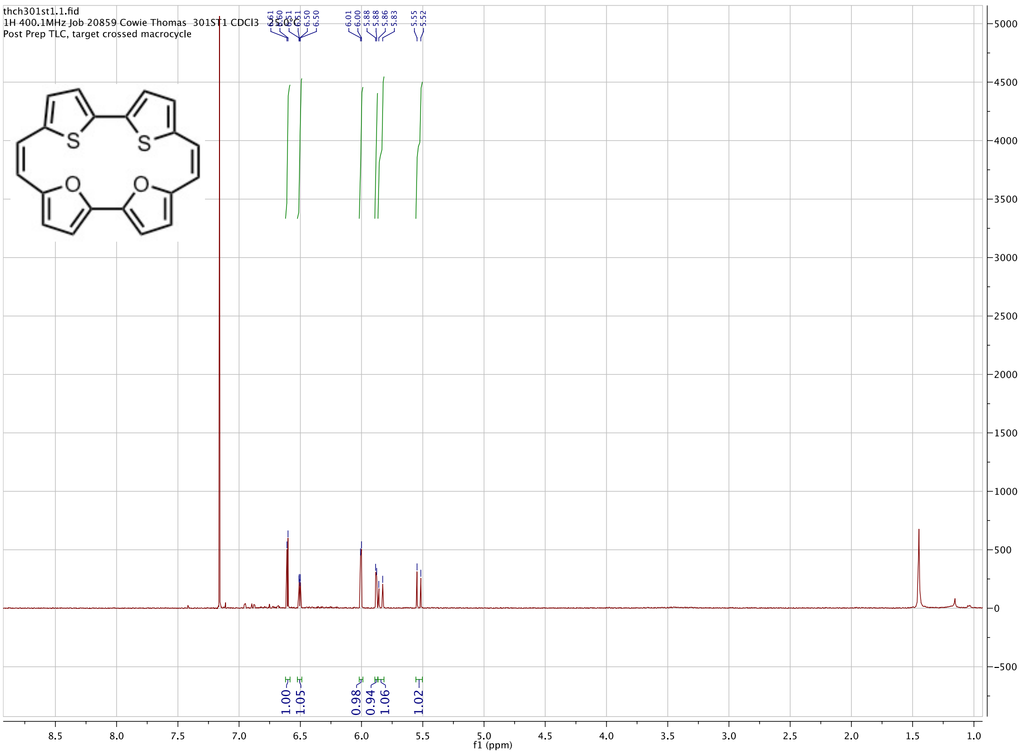

Figure 9. ^{1}H NMR Spectrum of 16 in $CDCl_3$

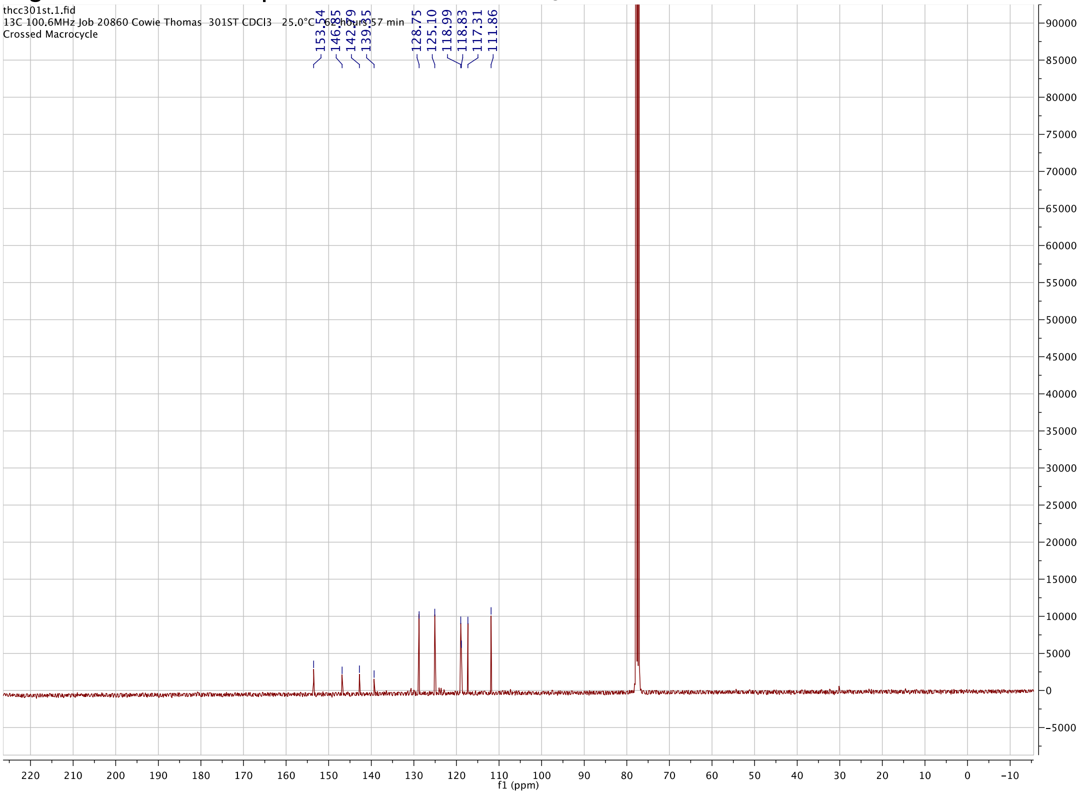

Figure 10. ^{13}C NMR Spectrum of 16 in $CDCl_3$

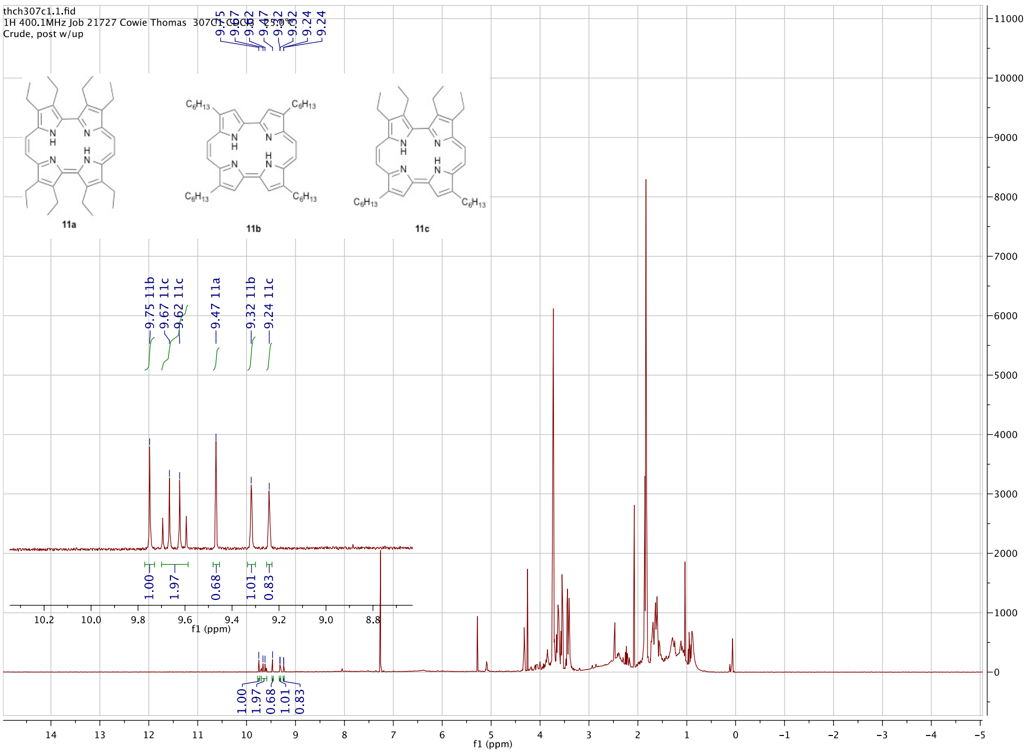

Figure 11. ^{1}H NMR spectra of crossed McMurry reaction between compound 6 & 7; Ratios of the products are shown, **11a (**20%), **11b** (26%), **11c** (54%, 5% isolated).

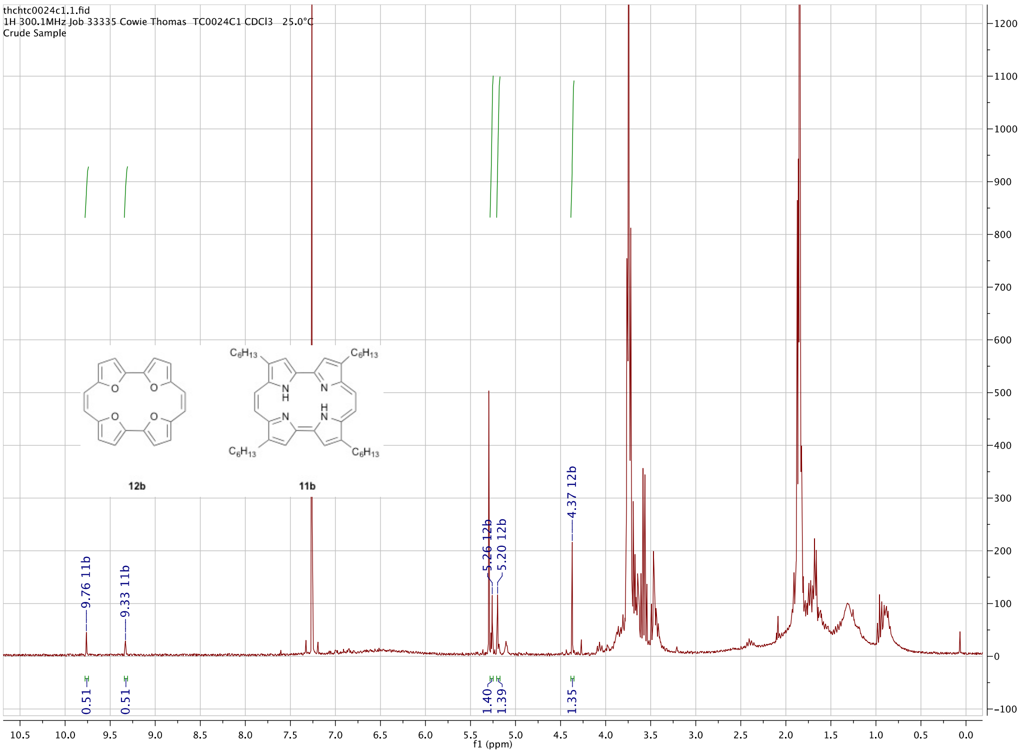

Figure 12. ^{1}H NMR spectra of crossed McMurry reaction between compound 6 & 9; Ratios of the products are shown, **11b** (27%), **12b** (73%).

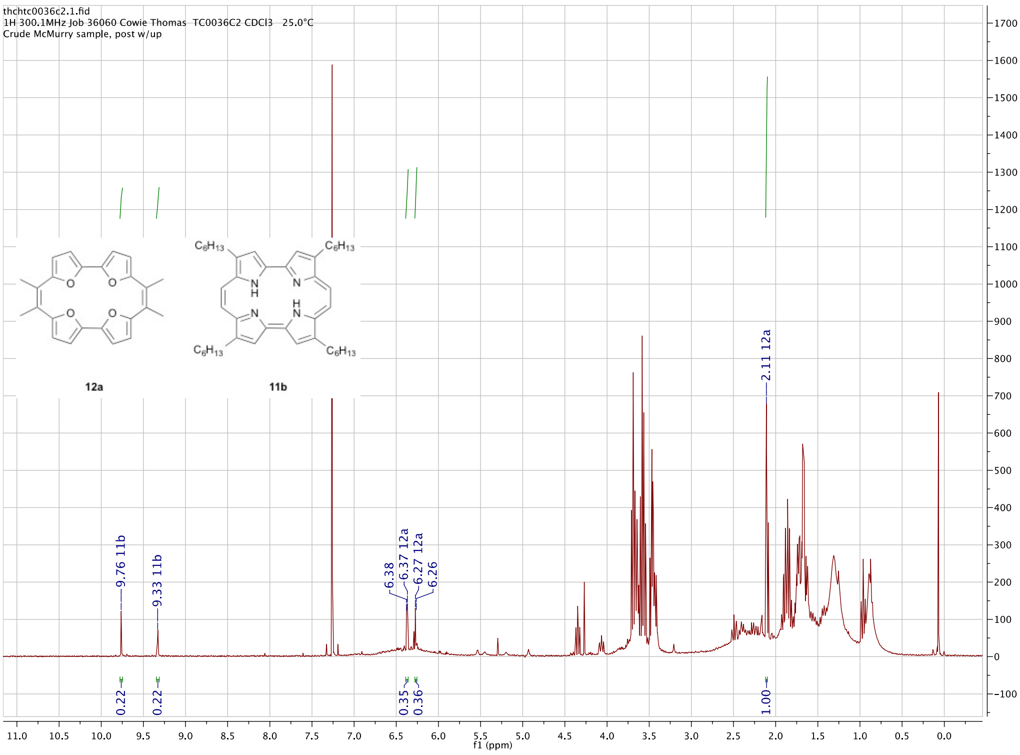

Figure 13. ^{1}H NMR spectra of crossed McMurry reaction between compound 6 & 10; Ratios of the products are shown, **11b** (41%), **12a** (59%).

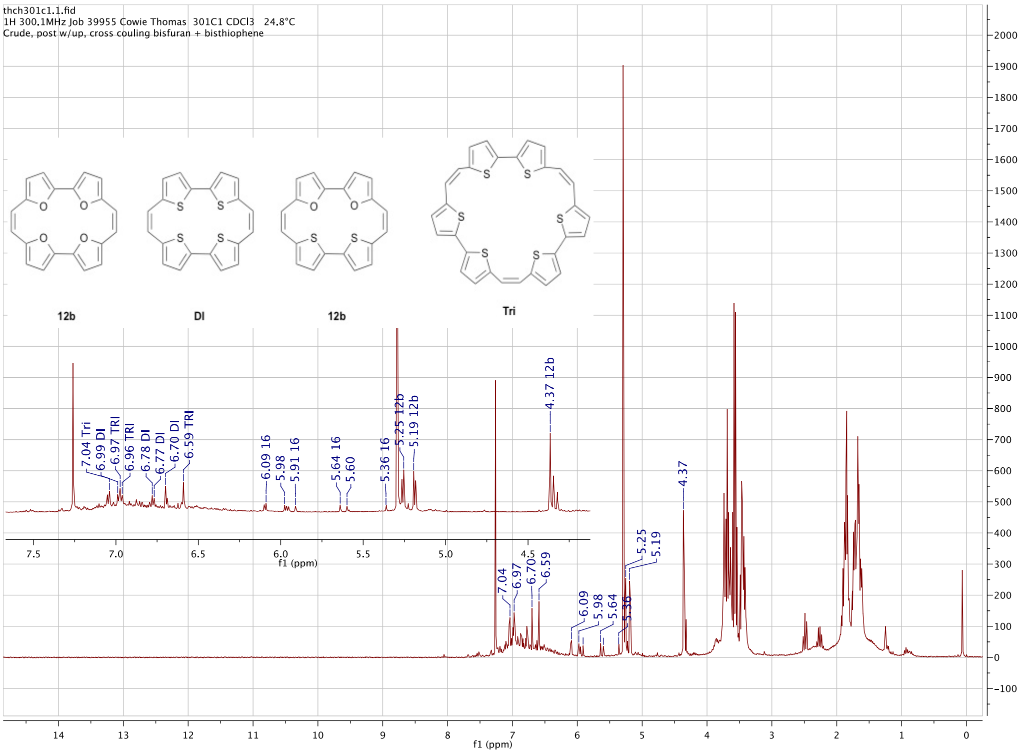

Figure 14. ^{1}H NMR spectra of crossed McMurry reaction between compound 9 & 15; Ratios of the products are shown, **12b** (45%), Bisthiophene Dimer (**DI**, 17%), Disthiophene Trimer (**TRI,** 14%), **16** (24%, 8% isolated).

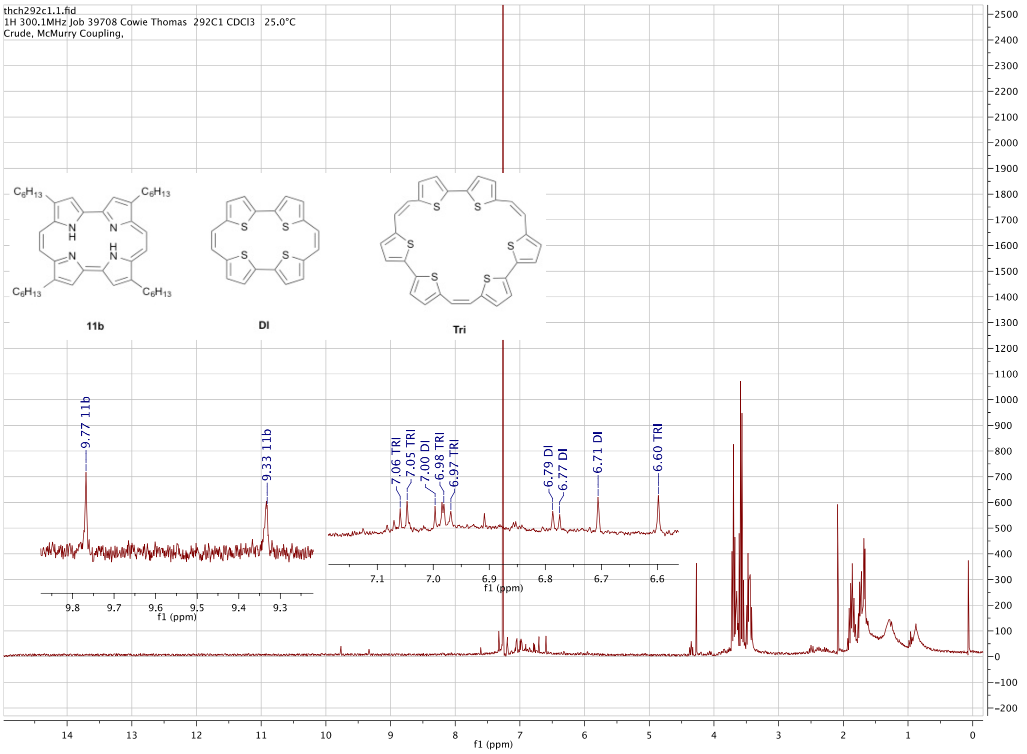

Figure 15. ^{1}H NMR spectra of crossed McMurry reaction between compound 6 & 15; Ratios of the products are shown, **11b** (36%), Bisthiophene Dimer (**DI**, 47%), Disthiophene Trimer (**TRI,** 18%).
